# A Novel *MMP12* Locus Is Associated with Large Artery Atherosclerotic Stroke Using a Genome-Wide Age-at-Onset Informed Approach

**DOI:** 10.1371/journal.pgen.1004469

**Published:** 2014-07-31

**Authors:** Matthew Traylor, Kari-Matti Mäkelä, Laura L. Kilarski, Elizabeth G. Holliday, William J. Devan, Mike A. Nalls, Kerri L. Wiggins, Wei Zhao, Yu-Ching Cheng, Sefanja Achterberg, Rainer Malik, Cathie Sudlow, Steve Bevan, Emma Raitoharju, METASTROKE, International Stroke Genetics Consortium, Wellcome Trust Case Consortium 2 (WTCCC2), Niku Oksala, Vincent Thijs, Robin Lemmens, Arne Lindgren, Agnieszka Slowik, Jane M. Maguire, Matthew Walters, Ale Algra, Pankaj Sharma, John R. Attia, Giorgio B. Boncoraglio, Peter M. Rothwell, Paul I. W. de Bakker, Joshua C. Bis, Danish Saleheen, Steven J. Kittner, Braxton D. Mitchell, Jonathan Rosand, James F. Meschia, Christopher Levi, Martin Dichgans, Terho Lehtimäki, Cathryn M. Lewis, Hugh S. Markus

**Affiliations:** 1 Stroke and Dementia Research Centre, St George's University of London, London, United Kingdom; 2 Department of Clinical Chemistry, Fimlab Laboratories, Tampere, Finland; 3 School of Medicine, University of Tampere, Tampere, Finland; 4 Center for Clinical Epidemiology and Biostatistics, School of Medicine and Public Health, University of Newcastle, Callaghan, New South Wales, Australia; 5 Hunter Medical Research Institute, New Lambton Heights, New South Wales, Australia; 6 Center for Human Genetic Research, Massachusetts General Hospital, Boston, Massachusetts, United States of America; 7 Program in Medical and Population Genetics, Broad Institute of Harvard and MIT, Cambridge, Massachusetts, United States of America; 8 Laboratory of Neurogenetics, National Institute on Aging, Bethesda, Maryland, United States of America; 9 Cardiovascular Health Research Unit, Department of Medicine, University of Washington, Seattle, Washington, United States of America; 10 Perelman School of Medicine, Division of Translational Medicine and Human Genetics, University of Pennsylvania, Philadelphia, Pennsylvania, United States of America; 11 Department of Medicine, University of Maryland School of Medicine, Baltimore, Maryland, United States of America; 12 Research and Development Program, Veterans Affairs Maryland Health Care System, Baltimore, Maryland, United States of America; 13 Department of Neurology and Neurosurgery, Brain Center Rudolf Magnus, University Medical Center Utrecht, Utrecht, The Netherlands; 14 Institute for Stroke and Dementia Research, Klinikum der Universität München, Ludwig-Maximilians-Universität, Munich, Germany; 15 Division of Clinical Neurosciences and Insititute of Genetics and Molecular Medicine, University of Edinburgh, Edinburgh, United Kingdom; 16 Clinical Neurosciences, University of Cambridge, Cambridge, United Kingdom; 17 Department of Surgery, Tampere University Hospital, Tampere, Finland; 18 KU Leuven - University of Leuven, Department of Neurosciences, Experimental Neurology - Laboratory of Neurobiology, Leuven, Belgium; 19 VIB - Vesalius Research Center, Leuven, Belgium; 20 University Hospitals Leuven, Department of Neurology, Leuven, Belgium; 21 Department of Clinical Sciences Lund, Neurology, Lund University, Lund, Sweden; 22 Department of Neurology and Rehabilitation Medicine, Skåne University Hospital, Lund, Sweden; 23 Department of Neurology, Jagiellonian University, Krakow, Poland; 24 School of Nursing and Midwifery, University of Newcastle, Callaghan, New South Wales, Australia; 25 Centre for Translational Neuroscience and Mental Health, University of Newcastle, Callaghan, New South Wales, Australia; 26 Institute of Cardiovascular and Medical Sciences, University of Glasgow, Glasgow, United Kingdom; 27 Julius Center for Health Sciences and Primary Care, University Medical Center Utrecht, Utrecht, The Netherlands; 28 Imperial College Cerebrovascular Research Unit (ICCRU), Imperial College London, London, United Kingdom; 29 Department of Cereberovascular Disease, Fondazione Istituto di Ricovero e Cura a Carattere Scientifico (IRCCS) Istituto Neurologico Carlo Besta, Milan, Italy; 30 Stroke Prevention Research Unit, Nuffield Department of Clinical Neuroscience, University of Oxford, Oxford, United Kingdom; 31 Department of Medical Genetics, University Medical Centre, Utrecht, The Netherlands; 32 Division of Genetics, Brigham and Women's Hospital, Harvard Medical School, Boston, Massachusetts, United States of America; 33 Department of Biostatistics and Epidemiology, University of Pennsylvania, Philadelphia, Pennsylvania, United States of America; 34 Center for Non-Communicable Diseases, Karachi, Pakistan; 35 Department of Neurology, Mayo Clinic, Jacksonville, Florida, United States of America; 36 Munich Cluster for Systems Neurology (SyNergy), Ludwig-Maximilians-Universität, Munich, Germany; 37 Department of Medical & Molecular Genetics, King's College London, London, United Kingdom; 38 Social, Genetic and Developmental Psychiatry Centre, Institute of Psychiatry, King's College London, London, United Kingdom; University of Exeter Medical School, United Kingdom

## Abstract

Genome-wide association studies (GWAS) have begun to identify the common genetic component to ischaemic stroke (IS). However, IS has considerable phenotypic heterogeneity. Where clinical covariates explain a large fraction of disease risk, covariate informed designs can increase power to detect associations. As prevalence rates in IS are markedly affected by age, and younger onset cases may have higher genetic predisposition, we investigated whether an age-at-onset informed approach could detect novel associations with IS and its subtypes; cardioembolic (CE), large artery atherosclerosis (LAA) and small vessel disease (SVD) in 6,778 cases of European ancestry and 12,095 ancestry-matched controls. Regression analysis to identify SNP associations was performed on posterior liabilities after conditioning on age-at-onset and affection status. We sought further evidence of an association with LAA in 1,881 cases and 50,817 controls, and examined mRNA expression levels of the nearby genes in atherosclerotic carotid artery plaques. Secondly, we performed permutation analyses to evaluate the extent to which age-at-onset informed analysis improves significance for novel loci. We identified a novel association with an *MMP12* locus in LAA (rs660599; p = 2.5×10^−7^), with independent replication in a second population (p = 0.0048, OR(95% CI) = 1.18(1.05–1.32); meta-analysis p = 2.6×10^−8^). The nearby gene, *MMP12*, was significantly overexpressed in carotid plaques compared to atherosclerosis-free control arteries (p = 1.2×10^−15^; fold change = 335.6). Permutation analyses demonstrated improved significance for associations when accounting for age-at-onset in all four stroke phenotypes (p<0.001). Our results show that a covariate-informed design, by adjusting for age-at-onset of stroke, can detect variants not identified by conventional GWAS.

## Author Summary

Ischaemic stroke places an enormous burden on global healthcare. However, the disease processes that lead to stroke are not fully understood. Genome-wide association studies have recently established that common genetic variants can increase risk of ischaemic stroke and its subtypes. In this study, we aimed to identify novel genetic associations with ischaemic stroke and its subtypes by addressing the fact that younger onset cases may have a stronger genetic component, and using this information in our analyses. We identify a novel genetic variant on chromosome 11 (rs660599), which is associated with increased risk of large artery stroke. We also show that mRNA expression of the nearest gene (*MMP12*) is higher in arteries with the disease process underlying large artery stroke (atherosclerosis). Finally, we evaluate our novel analysis approach, and show that our method is likely to identify further associations with ischaemic stroke.

## Introduction

Genome-wide association studies (GWAS) in ischaemic stroke have begun to identify the common genetic variants that confer risk of the disease. However, there is considerable heterogeneity present in stroke phenotypes: GWAS analyses have primarily looked at the three main subtypes; cardioembolic (CE), large artery atherosclerosis (LAA) and small vessel disease stroke (SVD). Within these subtype analyses, numbers of cases are smaller, but the expectation is that the effects of SNPs identified within the subtypes will be considerably larger. Indeed, all validated GWAS SNPs for ischaemic stroke to date have been stroke subtype-specific [1], [2], [3], [4], [5], indicating the importance of subtyping of cases.

Clinical risk factors are important in stroke; as many as 77% of first-ever stroke patients are hypertensive [6], and other factors such as diabetes mellitus and elevated serum cholesterol confer a considerable proportion of disease risk [7]. These risk factors increase in prevalence in older age groups, suggesting older stroke patients may have a reduced stroke-specific genetic contribution. Indeed, IS is uncommon in individuals below middle age, but increases greatly in prevalence beyond the age of 65 [8], with a lifetime risk of 1 in 5 for women and 1 in 6 for men [9].

Under the assumptions of the liability threshold model, the low prevalence of IS in younger age ranges suggests that individuals who do suffer strokes in this age group are likely to have an increased genetic predisposition. This is supported by family history data; with stronger family history seen in younger onset cases [10], [11], [12], and twin studies [13], which suggest that early onset cases may have higher heritability. We recently showed stronger effects for all stroke-associated SNPs in younger age groups, found evidence genome-wide that a significant number of SNPs show stronger association p-values when the oldest cases are removed, and showed increased pseudoheritability estimates for younger onset cases in certain stroke subtypes, thereby supporting this hypothesis [14]. However, the question of how best to integrate this information into GWAS analyses of ischaemic stroke remains unanswered. Previous GWAS have analysed younger subsets of ischaemic stroke cases [1], [15], but this approach may not be optimal for existing GWAS datasets if the increase in odds ratios for SNPs in younger cases are not sufficient to justify discarding a large proportion of the ascertained cases. All previous young onset analyses have been restricted to all ischaemic stroke cases versus controls; this may be particularly relevant given that all known loci for ischaemic stroke to date are for stroke subtypes [16].

A recent publication [17], outlined a novel method of informing genetic association analyses on important clinical covariates. Using the liability threshold model in conjunction with estimates of disease prevalence for individuals with specific clinical covariates, the method estimates posterior disease liabilities for each individual in a GWAS, and uses these liabilities in regression analyses to test for association with genome-wide SNPs. This approach avoids issues due to multiple testing across age-at-onset thresholds, and provides a simple solution that is rooted is previous epidemiological research. In the present study, we extend the clinical covariate informed analysis approach to imputed genotypes, informing our analyses on the age-at-onset to identify novel variants associated with IS. We perform a genome-wide analysis with four stroke phenotypes (IS, CE, LAA, SVD), and then determine the utility of the approach in ischaemic stroke GWAS, testing whether SNPs increase in significance.

## Results

### Association analysis

We performed age-at-onset informed association analysis for a total of 6,778 ischaemic stroke cases and 12,095 controls across four ischaemic stroke phenotypes; all IS and the three major subtypes: CE, LAA, and SVD (Table 1); with 1,637, 1,316, and 1,108 cases in the CE, LAA and SVD analyses respectively. With the exception of the young Milanese cohort, the age-at-onset distributions were similar in all cohorts (Table S3).

**Table 1:** Sample size of discovery populations.

Study Population	IS	CE	LAA	SVD	Controls
Belgium – Immunochip	396	147	57	49	319
Germany-Immunochip	421	127	101	-	2,355
Krakow – Immunochip	384	119	33	28	255
Sweden – Immunochip	796	246	56	183	997
UK – Immunochip	867	130	152	257	1,790
Germany – WTCCC2	1,174	330	346	106	797
UK – WTCCC2	2,374	474	498	460	5,175
Milano	366	64	73	25	407
Total (Discovery)	6,778	1,637	1,316	1,108	12,095

IS, all ischaemic stroke; CE, cardioembolic stroke; LAA, large artery stroke; SVD, small vessel disease.

**Figure 1:**
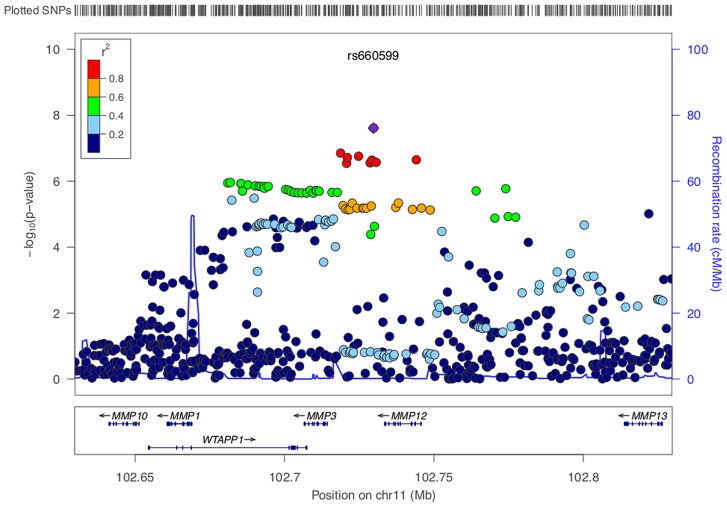
LocusZoom plot of *MMP12* association using age-at-onset informed approach. SNPs are colored based on their correlation (r^2^) with the labeled top SNP, which has the smallest P value in the region. The fine-scale recombination rates estimated from 1000 Genomes (EUR) data are marked in light blue, with genes marked below by horizontal blue lines. Arrows on the horizontal blue lines show the direction of transcription, and rectangles are exons. SNP p-values are from the discovery meta-analysis only with the exception of rs660599, for which the given p-value indicates the overall evidence for association from the discovery and replication cohorts.

We identified a group of twenty SNPs proximal to *MMP3* and *MMP12* on chromosome 11 in the LAA subtype that met our criteria for replication. The strongest associated of these was rs662558 (p = 1.4×10^−7^), a SNP that is in 1000 Genomes, but not HapMap II. Therefore, to enable replication in existing METASTROKE datasets, which were imputed to HapMap II, we selected the most strongly associated SNP from the HapMap II panel, which was in perfect LD with the lead SNP in our discovery meta-analysis (rs660599: uninformed, p = 1.6×10^−6^; informed, p = 2.5×10^−7^; Figure 1) [16]. We found no evidence of between-study heterogeneity at either SNP (Cochran's Q p = 0.22 and p = 0.19 for rs662558 and rs660599, respectively). The evidence of an age-at-onset effect at rs660599 was p = 0.011 (from permutations). We calculated age-at-onset quartiles for all large artery stroke cases from the discovery cohorts, and used these to evaluate this region at different age-at-onset thresholds. The median age-at-onset was 71 years, and the interquartile range was between 61 and 78 years. Post-hoc analyses of rs660599 in the discovery cohorts using logistic regression (full details in Text S2) showed considerably stronger associations in younger age-at-onset quantiles (Q1; OR(95% CI) = 1.83 (1.46–2.30), Q1–Q2; 1.56 (1.33–1.83), Q1–Q3; 1.30 (1.14–1.49), Q1–Q4; 1.30 (1.15–1.46)). No other regions met our criteria for replication.

### Replication analysis

The associated locus was evaluated in a further 1,881 large artery stroke cases and ancestry matched controls in 9 cohorts from METASTROKE (Table 2). We found evidence for replication of the SNP (rs660599) in all large artery stroke cases of European Ancestry (p = 0.0048, OR(95% CI) = 1.18(1.05–1.32)). Combining this result with the discovery p-value gave a genome-wide significant p-value of 2.6×10^−8^ (Table 3). Secondly, we used the Han and Eskin random effects meta-analysis approach to evaluate the association [18] after including a further 355 cases and 1,390 controls of Pakistani ancestry. The evidence for replication in this sample was p = 0.0063, giving an overall p-value of 3.4×10^−8^. Age-at-onset information was available across all age-at-onset quantiles for a subset of the replication studies (1,240 cases, 9,238 controls; ASGC, HVH, ISGS/SWISS, MGH-GASROS, Utrecht). We evaluated the SNP (rs660599) in these studies at different age-at-onset quantiles using logistic regression, meta-analysing as previously. We again found the strongest effects in the youngest age quantile, consistent with a stronger effect in younger onset cases (Q1; OR(95% CI) = 1.27(1.02–1.57), Q1–Q2; 1.18(1.00–1.39), Q1–Q3; 1.22(1.05–1.40), Q1–Q4; 1.22(1.07–1.41)).

**Table 2:** Sample size of replication populations.

Study Population	LAA (age<61)	LAA (age<71)	LAA (age<78)	LAA (all ages)	IS	Controls
ARIC	-	-	-	31	385	8,803
ASGC	81	179	277	421	1,162	1,244
deCODE	-	-	-	255	2,391	26,970
GEOS	-	-	-	37	448	498
HVH	18	39	63	71	566	2,072
ISGS/SWISS	84	130	179	217	1,070	1,370
MGH-GASROS (Affymetrix)	31	60	79	102	485	3,030
MGH-GASROS (Illumina)	22	47	59	68	296	377
PROMISe	134	230	301	324	556	1,145
RACE	-	-	-	355	1,390	5,308
Total (Replication)	370	685	958	1,881	8,749	50,817

LAA, large artery stroke; IS, all ischaemic stroke; ARIC, the Atherosclerosis Risk in communities study; ASGC, the Australian Stroke Genetics collaboration; deCODE, deCODE genetics; GEOS, the Genetics of early onset stroke study; HVH, the heart and vascular health study; ISGS/SWISS, the Ischaemic stroke genetics study/Siblings with Ischaemic stroke study; MGH-GASROS, Massachusetts General Hospital – Genetics affecting stroke risk and outcome; PROMISe, Prognostic modeling in ischaemic stroke study [55]; RACE, Risk Assessment of Cerebrovascular Events study. For further details of these populations please see the original METASTROKE publication [16].

**Table 3:** Evidence for association of A allele of rs660599 (chromosome 11; Base position 102,234,967) with large artery atherosclerotic stroke and all ischaemic stroke.

Subtype	SNP	RAF	p-value (discovery)	OR (95% CI) (EUR replication)	p-value (EUR replication, overall)	p-value (ALL replication, overall)
LAA	rs660599	0.19	2.5.×10^−7^	1.18 (1.05–1.32)	0.0048, 2.6×10^−8^	0.0063, 3.4×10^−8^
IS	“	“	3.2×10^−4^	1.05 (1.00–1.11)	0.050, 1.9×10^−4^	0.098, 3.6×10^−4^
CE	“	“	0.13	-	-	-
SVD	“	“	0.30	-	-	-

LAA, large artery stroke; IS, all ischaemic stroke; SNP, single nucleotide polymorphism; RAF, risk allele frequency; OR, odds ratio; 95% CI, 95% confidence interval; EUR, meta-analysis in individuals of European ancestry alone; ALL, trans-ethnic meta-analysis of all individuals. Forest plots of effect sizes and standard errors for each replication centre are given in Figures S3, S4.

### mRNA expression in carotid plaques

mRNA expression of the two proximal genes, *MMP3* and *MMP12* was analysed from 29 carotid, 15 abdominal aorta, 24 femoral plaques, and 28 atherosclerosis free left internal thoracic artery controls. *MMP12* expression was upregulated in carotid plaques compared with left internal thoracic artery controls (P = 1.2×10^−15^; fold change [FC] = 335.6). It was also upregulated in femoral plaques (P = 3.2×10^−14^; FC = 306.0) and abdominal plaques (P = 5.0×10^−11^; FC = 399.3) compared with controls. Conversely, *MMP3* was not significantly overexpressed in carotid, femoral or abdominal plaques versus controls (p>0.05).

### Regulatory information from ENCODE

Eight SNPs were identified that were perfect proxies (r^2^ = 1) with the associated SNP (rs660599) in the region. Seven of the SNPs were in an intergenic region between *MMP3* and *MMP12*, while one fell within an intron of *MMP12*. We investigated the evidence that any of these SNPs are functional variants using RegulomeDB [19]. Of the eight SNPs, we found strong evidence that one of these SNPs (rs586701) affects binding. The SNP overlaps both CHIP-seq and DNA-seq peaks from ENCODE analyses, indicating that there is open chromatin in the region, and therefore that the SNP is likely to be functional. There is also evidence from a separate CHIP-seq analysis that the SNP affects protein binding [20], and evidence from multiple sources that the SNP overlaps a predicted motif [21], [22], [23]. Histone modifications were observed in CHIP-seq experiments from ENCODE in a number of cells types, including Human umbilical vein endothelial (Huvec) cells. Two other SNPs (rs17368582, rs2276109) in moderate LD with the associated SNP (r^2^ = 0.64) have been previously shown to directly influence *MMP12* expression by affecting the affinity of an AP-1 binding site in the *MMP12* promoter region [24], [25]. Using RegulomeDB, we found further evidence from ENCODE that one of these SNPs (rs2276109) is indeed functional, giving evidence that the associated locus in this analysis is likely to affect *MMP12* expression through altered transcription. Detailed results for all analysed SNPs are given in Table S1. Additionally, we investigated if these SNPs (rs17368582, rs2276109, rs586701) were associated with *MMP12* expression in tissues from the GTEx project [26]. However, we could not confirm an association with *MMP12* expression in any relevant tissues (p>0.4 in whole blood, tibial artery, aortic artery).

### Evaluation of age-at-onset informed approach

Finally, we evaluated the overall utility of the age-at-onset informed approach in permutation analyses for SNPs that met p-value thresholds in the case control discovery data set. We generated 1000 permutations of age-at-onset within each centre, and performed age-at-onset informed analysis and subsequent meta-analysis for these SNPs, in the relevant stroke subtype.

**Figure 2:**
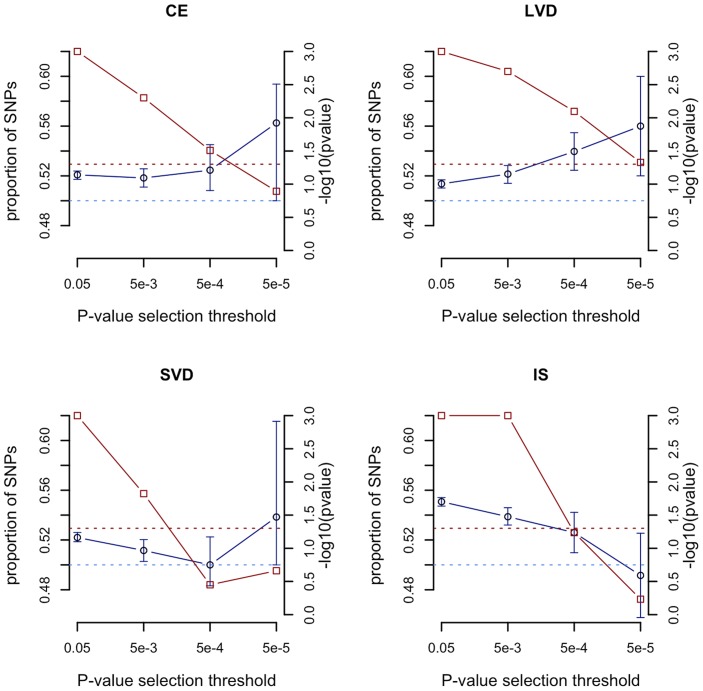
Evaluation of evidence genome-wide for SNPs exhibiting greater significance using the age-at-onset informed approach compared to permutations. -log10(p value) from permutations for evidence of age-at-onset effect at given SNP p-value selection threshold shown in red; median proportion of SNPs (with IQR) more significant in observed age-at-onset informed meta-analysis compared to permutations shown in blue; horizontal line at p = 0.05 in red; horizontal line at median proportion of SNP = 0.5 in blue; IS, all ischaemic stroke; CE, cardioembolic stroke; LAA, large artery atherosclerotic stroke; SVD, small vessel disease. See Table S5 for number of SNPs included at each p-value selection threshold.

We compared the sum of the meta-analysis Z scores from all SNPs with p<0.05 in the observed age at onset informed meta-analysis with those from permutations. At this p-value selection threshold, we found strong evidence (p<0.001) for genome-wide age-at-onset effects in each of the stroke phenotypes, with consistently increased summed Z scores in the observed age-at-onset informed meta-analysis compared to the permutations (Figure 2, red points, right hand axis). These results suggest that many of the risk variants for each stroke subphenotype have a higher frequency in younger onset cases. As the p-value selection threshold decreased, the summed Z score statistic became less significant in each stroke type, possibly reflecting lower overall power when fewer SNPs are included, even as these SNPs may have larger average effects. Further details are seen from the median proportion of SNPs more significant in the age-at-onset informed analysis than in the permutations (Figure 2, blue points, left hand axis). For CE and LAA stroke, the proportions increased with more stringent p-value thresholds (from 52.1% to 56.3% for p<0.05 and p<0.00005 thresholds in CE, and from 51.4% to 56.0% for p<0.05 and p<0.00005 thresholds in LAA). Interestingly, in the all ischaemic stroke analysis the median proportion of SNPs more significant in the observed results than permutations dropped from 55.1% for SNPs with p<0.05 to 49.2% for only SNPs with p<0.00005. This result may indicate a reduced proportion of true associations at stricter p-value thresholds for all ischaemic stroke compared to the subtypes, which is consistent with the observation that all common variants associated with stroke are for stroke subtypes, rather than for the phenotype of all ischaemic stroke [16].

**Figure 3:**
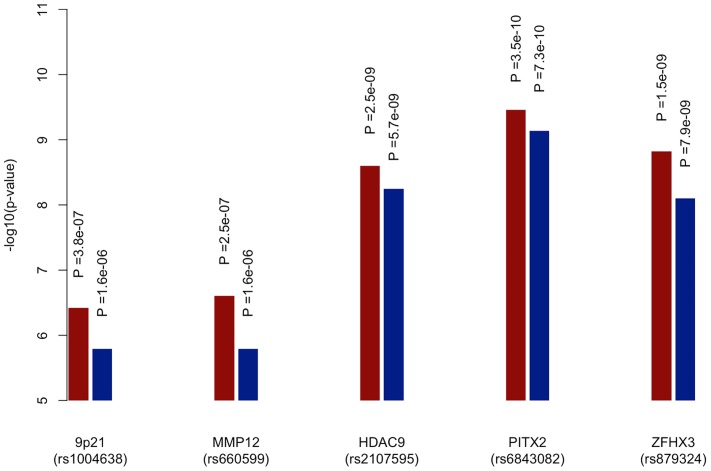
Meta-analysis p-values of known loci for ischaemic stroke subtypes using age-at-onset informed approach compared to uninformed approach. -log10 of p-values derived from meta-analysis of all discovery cohorts using age-at-onset informed approach (red) and uninformed approach (blue). 9p21 (rs1004638), *MMP12* (rs660599) and *HDAC9* (rs2107595) p-values calculated within large artery atherosclerosis subtype of stroke, *PITX2* (rs6843082) and *ZFHX3* (rs879324) p-values calculated with cardioembolic stroke subtype.

The previously reported GWAS associations from a recent ischaemic stroke meta-analysis (9p21, *HDAC9*, *PITX2*, *ZFHX3*) were all found to be more significant using the age-at-onset informed approach than the uninformed analysis (Figure 3). The increase in significance ranged from over half an order of magnitude (7.9×10^−9^ to 1.5×10^−9^ for rs879324 in *ZFHX3*, CE), to under half an order of magnitude (5.7×10^−9^ to 2.5×10^−9^ for rs2107595 in *HDAC9*, LVD). To ensure these analysis methods were comparable, we calculated genomic inflation factors and plotted QQ-plots. These were similar in the standard and the age-at-onset informed approach (Table S4, Figure S1, S2). For these four associated SNPs, we further used the permuted data sets to assess the observation of increased significance in the age-at-onset informed analysis. We compared the observed meta-analysis p-value to those from the permutations, generating an empirical p-value by dividing the number of permutations more significant than the observed results by the number of permutations. In LAA stroke, we observed a significant age-at-onset effect (p = 0.018, 0.011 and 0.002 for the *HDAC9*, *MMP12* and 9p21-associated SNPs in Figure 3, respectively). Similarly, for CE, we observed a significant age-at-onset effect for rs879324 (*ZFHX3*, p = 0.026), and a near-significant effect in rs6843082 (*PITX2*, p = 0.081). This result provides further evidence that risk variants associated with ischaemic stroke subtypes have a stronger role in younger onset cases, and suggests that the age-at-onset informed approach will produce improved significance when the magnitude of genetic effects are stronger in younger onset cases.

## Discussion

We used a large GWAS dataset to evaluate the utility of an age-at-onset informed analysis approach to ischaemic stroke, and to identify novel variants associated with ischaemic stroke phenotypes. We identified a novel *MMP12* locus that is associated with large artery atherosclerotic stroke, and verified that the age-at-onset informed approach produces improved significance for loci associated with each of the stroke phenotypes studied, as well as demonstrating that it increased the significance of four previous GWAS associations with ischemic stroke, all without systematic inflation of the test statistic. Importantly, the novel associated SNP would not have been identified using a standard logistic regression framework.

We identified a group of SNPs proximal to Matrix Metalloproteinase 12 (*MMP12*) that showed increased significance when using the age-at-onset informed approach. The increase in significance from the equivalent uninformed analysis was of almost an order of magnitude (from p = 1.6×10^−6^ to p = 2.5×10^−7^ for rs660599). We took a single SNP from this region forward for replication in an independent dataset, finding further evidence that the region is associated with large artery stroke. Two SNPs (rs17368582, rs2276109) in this LD-block have previously been shown to directly influence *MMP12* expression by affecting the affinity of an AP-1 binding site in the *MMP12* promoter region [24], [25], and another variant in this block (rs17361668) is associated with increased fibrinogen levels, leading to an increased risk of developing advanced carotid atherosclerotic lesions, and an increased risk of myocardial infarction. We identified a second functional candidate (rs586701), which falls within both CHIP-seq and DNA-seq peaks from ENCODE, and is in complete LD with the associated SNP in our analysis.

We investigated mRNA expression of *MMP12* and *MMP3* in carotid atherosclerotic plaques in individuals from the Tampere Vascular Study. *MMP12* was overexpressed in diseased tissue compared to healthy controls, while no significant difference was found for the other nearby gene, *MMP3*. *MMP12* is a member of the Matrix Metalloproteinase (MMP) family of proteases, which are capable of degrading extracellular matrix proteins, and have a prominent role in atherosclerosis. They are thought to promote macrophage invasion [27], [28], [29], promote angiogenesis [30], and show increased activity in atheromatous plaques [31]. *MMP12* deletions are associated with smaller, more stable lesions in the brachiocephalic artery of rabbits [32], and reduced elastin degradation in the aortic arch [33], indicating that *MMP12* may have a role in destabilising plaques. Studies in humans have found *MMP12* is localized to the core of advanced plaques, in macrophages with decreased arginase-I expression [34], that *MMP12* localizes selectively to macrophages at the borders of the lipid core [35], and that *MMP12* is significantly overexpressed in ruptured plaques when compared with thick or thin cap plaques, or with plaques with pathological intimal thickening [36]. This indicates that *MMP12* is likely be involved in late-stage plaque instability: our study suggests that genetic variation impacts on this process.

Secondly, we performed extensive permutation analyses to assess the utility of the age-at-onset informed approach genomewide. In each phenotype studied we found evidence that SNPs were more strongly associated using the approach than would be expected by chance, indicating that multiple risk variants are likely to be more common in younger onset cases. The significance was strongest when more SNPs were included in the analysis, which likely reflects the cumulative impact of age-at-onset effects on many SNPs. An alternative explanation might be that the increased significance for lower p-value thresholds is the result of the cumulative effects of subtle confounding. However, this is unlikely because any subtle biases will also be present in the permutations, and should therefore not affect the significance of the results. This result supports observations from family history and prospective cohort studies, which have observed stronger effects in younger onset cases [6], [11]. Furthermore, all known associations with stroke were more significant using the age-at-onset informed approach. The increase in significance was around half an order of magnitude (e.g from p = 7.9×10^−9^ to 1.5×10^−9^ for *ZFHX3*, Figure 2), and was significant in all but one locus, as assessed by permutation. Taken together, these results indicate that age-at-onset is an important measure to stratify stroke cases, and show that, as expected by theory [17], integrating this information into association studies is likely to increase power to identify novel loci when the relative contribution of genetic is dependent on age-at-onset.

Our study has limitations. We used imputed data from the Immunochip platform, meaning we only had access to ∼40% of the genome across all centres. Secondly, cases were drawn from a number of international centres, meaning that despite efforts to standardize phenotyping, we cannot rule out differences in screening and clinical ascertainment.

Of complex diseases, IS has a particularly large degree of heterogeneity, exemplified by the fact that all validated associations identified to date have been within subtypes defined by clinical and radiological information. Further heterogeneity by risk factor and clinical covariate profiles is likely to exist, but the optimal method of incorporating this information into analyses remains an unanswered question. Our results indicate that a covariate-informed design, conditioning on age-at-onset of stroke, can unearth further associated variants. We provide evidence for this by identifying an association with a novel *MMP12* locus in large artery stroke, supported by increased mRNA expression of the implicated gene in carotid plaques. GWAS in ischaemic stroke have begun to identify the genetic component of the disease, but these results are not yet clinically useful. Our study suggests that a more refined approach to analysis of genetic data, incorporating covariate information, is an important step in this process, and will help to ensure success in future GWAS.

## Materials and Methods

### Ethics statement

All studies were approved by their local ethics committees; all patients gave informed consent.

### Description of datasets

The initial dataset consisted of 6,778 ischaemic stroke cases of European ancestry and 12,095 ancestry-matched controls from the Wellcome Trust Case-Control Consortium II project in ischaemic stroke [1], as well as a cohort from Milan, Italy [16]. These included 2,858 cases and 5,716 matched controls genotyped using the Immunochip platform; and 3,940 cases genotyped using either the Illumina 610 k or 660 k platforms matched with 6,379 controls genotyped on the Illumina Human 1.2M Duo (UK), Illumina Human 550 k (German) and Illumina 610 k platforms (Italian) (Table 1). The Immunochip cases were described in the previous WTCCC2 ischaemic study, where they formed the replication effort [1], as well as in a recent paper [37]. Genotyping of the five Immunochip case cohorts on the commercially available Immunochip array (Illumina, San Diego, CA, USA) was performed at the Sanger Centre, Hinxton, Cambridge UK. Swedish controls were provided and genotyped by the Swedish SLE network, Uppsala, Sweden. Belgian control samples were provided through the efforts of the International Multiple Sclerosis Genetics Consortium (IMSGC). German controls were derived from the PopGen biobank, [38]. UK controls were derived from the 1958 Birth cohort. Any of the 1958 Birth controls overlapping with those from the WTCCC2 datasets, as assessed by IBD estimates, were removed prior to analysis. Standard quality control procedures were undertaken on all centres, before centre-wise imputation to the 1000 Genomes phase 1 integrated variant set (March 2012), using IMPUTE v2.2.0 [39], [40]. SNPs with poor imputation quality (info<0.3) or low minor allele frequency (MAF<0.01) were discarded.

Ischemic stroke was defined as a typical clinical syndrome with radiological confirmation; ascertained cases were classified into individual stroke subtypes using the Trial of Org 10172 in acute stroke (TOAST) criteria in all centres [41]. Age-at-onset was defined as age at first hospital admission for stroke; where this information was unavailable, age at blood draw was used (7.3% of cases). The age-at-onset and gender distributions of the populations are given in Table S3. Age-at-onset quantiles were calculated from all the cases from the discovery datasets in the four stroke phenotypes (all IS and the three stroke subtypes: CE, LAA, SVD) and these were used to evaluate associated loci at different age-at-onset thresholds.

### Association analysis

The prevalence of ischaemic stroke by age was obtained from a recent publication [9]; gender-specific estimates were averaged, and prevalences within each of the stroke subtypes were assumed to be approximately 20% of the overall total, similar to proportions seen in population-based studies [42]. We modeled phenotype data using a continuous unobserved quantitative trait called the disease liability, which we used to approximate the effect of age-at-onset on the liability scale, based on estimates of ischaemic stroke prevalence by age from epidemiological data (full details in Text S2). We developed two models for our analysis; one based on the prevalence rates for all ischaemic stroke cases, and secondly for the three stroke subtypes. We used these models to calculate posterior mean liabilities after conditioning on age-at-onset for the four stroke phenotypes separately. Controls were modeled in the same way, but were assumed to take the posterior mean from the lower (unaffected) portion of the distribution in the liability threshold model. Where age data was missing, individuals were assigned the median age value. Full descriptions of the models used and the formulae used to calculate posterior mean liabilities are given in Text S2. Regression was then performed on posterior liabilities by multiplying the number of samples by the squared correlation between the expected genotype dosage and posterior mean liabilities for each of the discovery cohorts in the four ischaemic stroke phenotypes (CE, LAA, SVD, IS), following a previous approach [17]. Ancestry-informative principal components were included where appropriate (6 of 8 centres), using the EIGENSTRAT procedure [43]. All analysis was performed using the R statistical software.

The results from each centre were meta-analysed for each of the four phenotypes using Stouffer's Z-score weighted approach, as implemented in METAL [44]. Genomic control was used to correct for any residual inflation due to population stratification [45]. Between-study heterogeneity was assessed using Cochran's Q statistic. We considered only SNPs present in at least 75% of the cases, and with no evidence of heterogeneity (Cochran's Q p-value>0.001). All SNPs analysed were either genotyped or imputed in both the Immunochip and the genome-wide datasets. After meta-analysis, the resulting p-values were compared with the equivalent values from an unconditioned analysis. For SNPs more significant in the age-at-onset informed analysis and with p<5×10^−6^, we determined the evidence of a true age-at-onset effect by generating 1000 permutations of age-at-onset and rerunning the age-at-onset informed analysis, meta-analysing as previously. We calculated an empirical p-value by dividing the number of permuted observations showing greater significance in the meta-analysis than the observed results by the number of permutations. Any novel SNP with a meta-analysis p<5×10^−6^ and evidence of an age-at-onset effect at p<0.05 were taken forward for replication. We set the experiment-wide significance threshold at p<5×10^−8^.

### Replication analysis

Replication of an associated variant was performed in a further 10 cohorts from METASTROKE. Nine of the centres used a cross-sectional design, while one was a large prospective, population based cohort (ARIC). Nine of the centres were of European ancestry, while one consisted of individuals of Pakistani ancestry (RACE) (Table 2). All centres used a case-control methodology; centres with a cross sectional design used logistic regression to model the association of genotype dosages from imputation with the dichotomous outcome of ischaemic stroke and prospective cohorts used Cox proportional-hazards models to evaluate time to first stroke, fitting an additive model relating genotype dose to the stroke outcome. European ancestry replication centres were meta-analysed using a fixed effects inverse-variance weighted method. To assess the evidence for association of the SNP for replication samples of all ancestries, we performed a trans-ethnic meta-analysis using a random-effects model to control for any resulting heterogeneity [18]. To evaluate the overall evidence for association, the results of the discovery and replication analyses were combined using Fisher's Method.

### mRNA expression in carotid atherosclerotic plaques

Expression of the two genes proximal to the associated variant was tested in atherosclerotic plaques from the Tampere Vascular study [27], [46], [47], [48], [49]. Carotid, femoral, and aortic atherosclerotic plaques constituting the intima and inner media were prospectively obtained between 2005 and 2009 from patients fulfilling the following inclusion criteria: (1) carotid endarterectomy attributable to asymptomatic or symptomatic >70% carotid stenosis, or (2) femoral or (3) aortic endarterectomy with aortoiliac or aortobifemoral bypass attributable to symptomatic peripheral arterial disease. Whole thickness left internal thoracic artery samples obtained during coronary artery bypass surgery and identified as being microscopically atherosclerosis free were used as controls. The patients were consecutively recruited and stratified according to indication for surgery. All open vascular surgical procedures were performed at the Division of Vascular Surgery and Heart Center, Tampere University Hospital.

Fresh tissue samples were immediately soaked in RNALater solution (Ambion Inc) and homogenized using an Ultra-Turrax T80 homogenizer (IKA). RNA was extracted with the Trizol reagent (Invitrogen) and miRNEasy Mini-Kit (Qiagen) with the RNase-Free DNase Set (Qiagen) according to manufacturer instructions. The RNA isolation protocol was validated by analyzing the integrity of the RNA with the RNA 6000 Nano Chip Kit (Agilent). The expression levels were analyzed with an Illumina HumanHT-12 v3 Expression BeadChip (Illumina). In brief, 300–500 ng of RNA was reverse transcribed in cRNA and biotin-UTP labeled using the IlluminaTotalPrep RNA Amplification Kit (Ambion), and 1500 ng of cRNA was then hybridized to the Illumina HumanHT-12 v3 Expression BeadChip.

The BeadChips were scanned with the Illumina iScan system. After background subtraction, raw intensity data were exported using the Illumina Genome Studio software. Further data processing was conducted by means of R language and appropriate Bioconductor modules. Data were log2-transformed, and robust multichip average and robust spline normalization (rma_rsn) were used. Accuracy of the expression array was validated with qRT-PCR [50]. mRNA Expression levels in the tissues were determined; a fold change statistic was estimated between the two tissues, and significance was calculated using a t test.

### Regulatory information using RegulomeDB

Recent evidence indicates that a significant proportion of GWAS SNPs fall within regions that are likely to affect binding of nearby proteins, such as transcription factor binding sites [51], [52]. We used the RegulomeDB database to access regulatory information from ENCODE and other existing publications [19], investigating the evidence that the SNPs in the associated locus have a regulatory function. First, the linkage-disequilibrium (LD) patterns amongst the most strongly associated SNPs were determined. We then used PLINK to determine the LD structure of the associated region, using LD-patterns from the 85 Utah residents from the 1000 Genomes project [53], [54]. All SNPs with r^2^>0.6 were identified within a 2,000 kb window from the index SNP. All of the SNPs identified were then investigated using RegulomeDB to determine the evidence that any of the SNPs have a regulatory function.

### Evaluation of age-at-onset informed approach

Permutation analysis was performed to evaluate the age-at-onset informed approach, to show that including age at onset information directly led to the increased significance, due solely to inclusion of age-at-onset information at tested SNPs. First, we identified a set of SNPs enriched for true association in the case control analysis of ischaemic stroke and subtypes. An expanded set of discovery and METASTROKE studies were analysed using standard case control methods and subsequent meta-analysis (see Table S2). SNPs with p<0.05 and no evidence of heterogeneity (p>0.0001) were extracted and pruned for LD (300 kb window, r^2^<0.25), leaving a set of almost independent SNPs for further analysis. Each retained SNP represented the most significant association in each LD block, as determined by the “clump” procedure in PLINK, based on LD patterns from the CEU individuals from 1000 Genomes. The number of SNPs used in each analysis is given in Table S5. These SNP subsets were derived for ischaemic stroke, and for each stroke subset and then used in the age-at-onset informed analysis. Analysis was performed as previously for each stroke subtype using the age-at-onset informed method within studies and meta-analysis across studies (giving *observed* results, as obtained above). We then performed a permutation study to obtain the expected distribution of p-values at these SNPs. Age at onset for cases was permuted within stroke subtypes within each study, and then the data were re-analysed, for 1000 permutations. Two summary statistics were constructed: (1) within permutations, we compared p-values from analysis of permuted age at onset with p-values from the observed data, and tabulated the proportion of SNPs with increased significance in the observed data set than in the permuted data set; across permutations, we calculated the median proportion of SNPs with increased significance in the observed data; (2) Within permutations, we converted each SNP p-value to a Z score and summed the absolute value of the Z score across SNPs (sumZ). An empirical p-value for the age-informed analysis was calculated from the proportion of simulated data sets where sumZ exceeded the value in the observed analysis. This analysis was performed at SNP subsets defined from four SNP p-value thresholds in the discovery and METASTROKE studies: p<0.05, p<0.005, p<0.0005, and p<0.00005.

Finally, we assessed the evidence of an age-at-onset effect at the four stroke loci identified in the METASTROKE ischaemic stroke collaboration (9p21, *HDAC9*, *PITX2*, *ZFHX3*) [16]. For each SNP, we generated an empirical p-value from the proportion of permutations showing stronger association than in the observed age-at-onset informed analysis.

## Supporting Information

Figure S1 QQ-plots for cardioembolic stroke and all ischaemic stroke analyses. QQ-plots of expected p-values (x-axis) against observed p-values (y-axis) for analyses of (clockwise from top left) cardioembolic stroke (age-at-onset informed), cardioembolic stroke (uninformed), all ischaemic stroke (uninformed), all ischaemic stroke (age-at-onset informed). Lambda values for each plot are given in Table S4.
(DOCX)

Figure S2 QQ-plots for large artery atherosclerotic stroke and small vessel disease stroke analyses. QQ-plots of expected p-values (x-axis) against observed p-values (y-axis) for analyses of (clockwise from top left) large artery stroke (age-at-onset informed), large artery stroke (uninformed), small vessel stroke (uninformed), small vessel stroke (age-at-onset informed). Lambda values for each plot are given in Table S4.
(DOCX)

Figure S3 Forest plot of SNP effects for rs660599 in the large artery atherosclerotic stroke replication populations. ASGC, the Australian Stroke Genetics collaboration; deCODE, deCODE genetics; GEOS, the Genetics of early onset stroke study; HVH, the heart and vascular health study; ISGS/SWISS, the Ischaemic stroke genetics study/Siblings with Ischaemic stroke study; MGH-GASROS, Massachusetts General Hospital – Genetics affecting stroke risk and outcome. PROMISe, Prognostic modeling in ischaemic stroke study; RACE, Risk Assessment of Cerebrovascular Events study.
(DOCX)

Figure S4 Forest plot of SNP effects for rs660599 in the large artery atherosclerotic stroke replication populations for cases with age <61 years. ASGC, the Australian Stroke Genetics collaboration; HVH, the heart and vascular health study; ISGS/SWISS, the Ischaemic stroke genetics study/Siblings with Ischaemic stroke study; MGH-GASROS, Massachusetts General Hospital – Genetics affecting stroke risk and outcome. PROMISe, Prognostic modeling in ischaemic stroke study.
(DOCX)

Table S1 Results from RegulomeDB, showing the evidence that SNPs in the associated *MMP12* region have a regulatory function. Scores indicate the following degrees of evidence: Score 2b, TF binding + any motif + DNase Footprint + DNase peak; Score 4, TF binding + DNase peak; Score 5, TF binding or DNase peak; Score 6, other; “No data” indicates that RegulomeDB holds no information about the given SNP, meaning there currently exists no evidence to suggest that the SNP has a regulatory function. In some cases this may indicate that the SNP falls within a protein-coding region. SNP, single nucleotide polymorphism.
(DOCX)

Table S2 Expanded set of populations used to generate SNPs with p<0.05 to evaluate the age-at-onset informed approach. ARIC, The Atherosclerosis Risk in Communities study; ASGC, Australian Stroke Genetics Collaborative; CHS, Cardiovascular Health Study; FHS, Framingham Heart Study; HPS, Heart Protection Study; HVH, The Heart and Vascular Health Study; ISGS/SWISS, The Ischemic Stroke Genetics Study/Sibling with Ischaemic Stroke Study; MGH-GASROS, The MGH Genes Affecting Stroke Risk and Outcome Study; WTCCC2-Germany, The Wellcome Trust Case-Consortium II Munich; WTCCC2-UK, The Wellcome Trust Case-Consortium II UK; RACE, Risk Assessment of Cerebrovascular Events Study, Pakistan.
(DOCX)

Table S3 **Age and gender distributions of populations.** ARIC, The Atherosclerosis Risk in Communities study; ASGC, Australian Stroke Genetics Collaborative; CHS, Cardiovascular Health Study; FHS, Framingham Heart Study; HPS, Heart Protection Study; HVH, The Heart and Vascular Health Study; ISGS/SWISS, The Ischemic Stroke Genetics Study/Sibling with Ischaemic Stroke Study; MGH-GASROS, The MGH Genes Affecting Stroke Risk and Outcome Study; WTCCC2-Germany, The Wellcome Trust Case-Consortium II Munich; WTCCC2-UK, The Wellcome Trust Case-Consortium II UK; RACE, Risk Assessment of Cerebrovascular Events Study, Pakistan. IS, all ischaemic stroke; CE, cardioembolic stroke; LAA, large artery stroke; SVD, small vessel disease.
(DOCX)

Table S4 Genomic inflation (λ) rates for discovery populations for age-at-onset informed and uninformed approaches. IS, all ischaemic stroke; CE, cardioembolic stroke; LAA, large artery stroke; SVD, small vessel disease.
(DOCX)

Table S5 Number of SNPs used in evaluation of age-at-onset informed approach. IS, all ischaemic stroke; CE, cardioembolic stroke; LAA, large artery stroke; SVD, small vessel disease.
(DOCX)

Text S1 Membership of Wellcome Trust Case Control Consortium 2 (WTCCC2).
(DOCX)

Text S2 Liability threshold models.
(DOCX)
