# Supplementary material for: A Novel MMP12 Locus Is Associated with Large Artery Atherosclerotic Stroke Using a Genome-Wide Age-at-Onset Informed Approach
Source: PLoS Genet. 2014 Jul 31;10(7):e1004469. doi: 10.1371/journal.pgen.1004469 (PMC4117446; doi:10.1371/journal.pgen.1004469)
Supplement: Figure S2 — QQ-plots for large artery atherosclerotic stroke and small vessel disease stroke analyses. QQ-plots of expected p-values (x-axis) against observed p-values (y-axis) for analyses of (clockwise from top left) large artery stroke (age-at-onset informed), large artery stroke (uninformed), small vessel stroke (uninformed), small vessel stroke (age-at-onset informed). Lambda values for each plot are given in Table S4. (DOCX) [file pgen.1004469.s002.docx]

**Figure S2 – QQ-plots for large artery atherosclerotic stroke and small vessel disease stroke analyses**


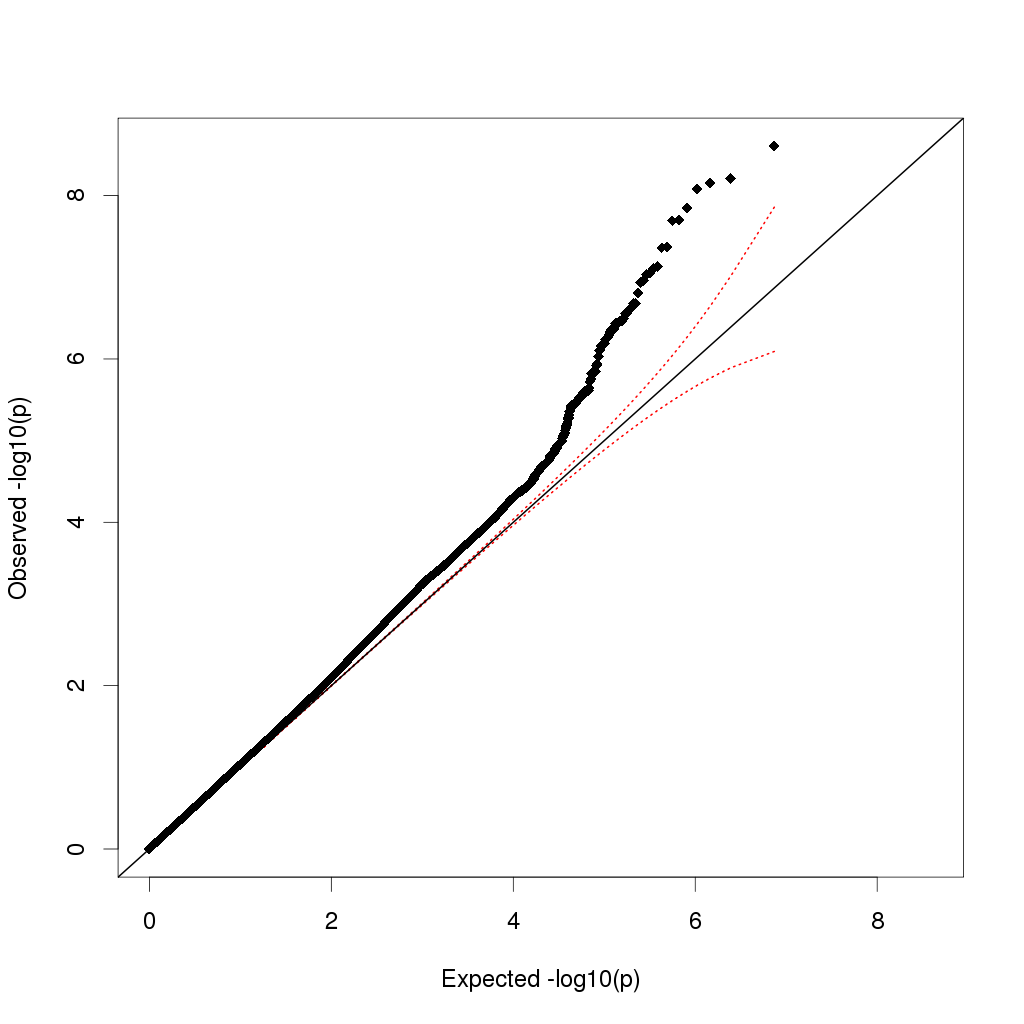

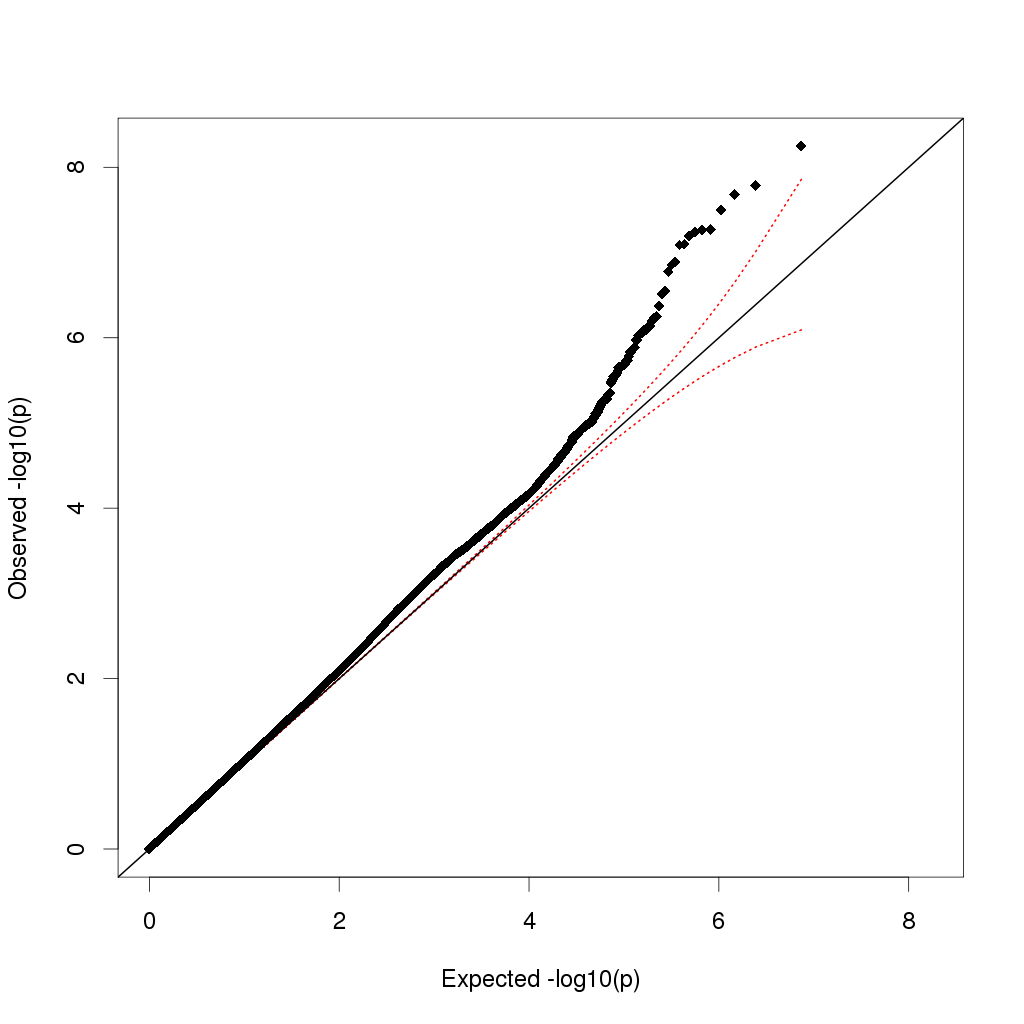

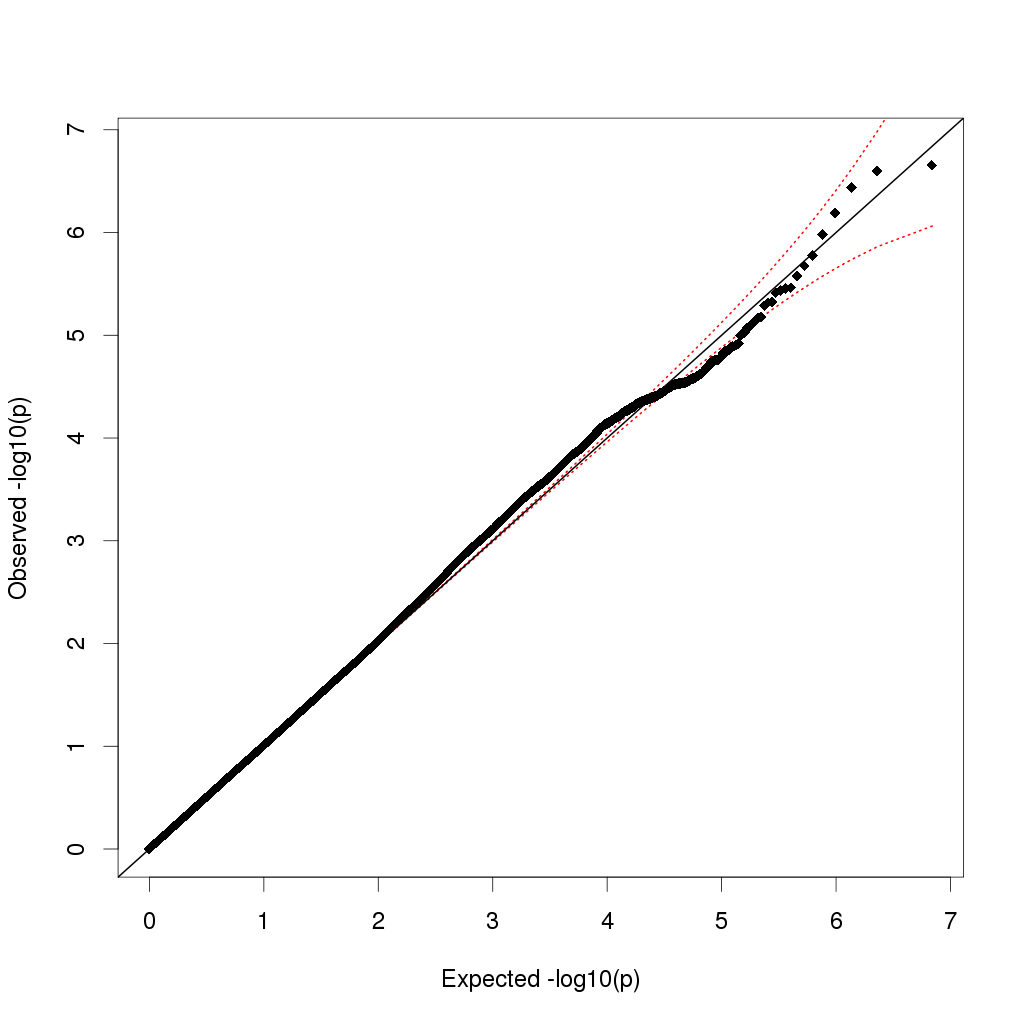

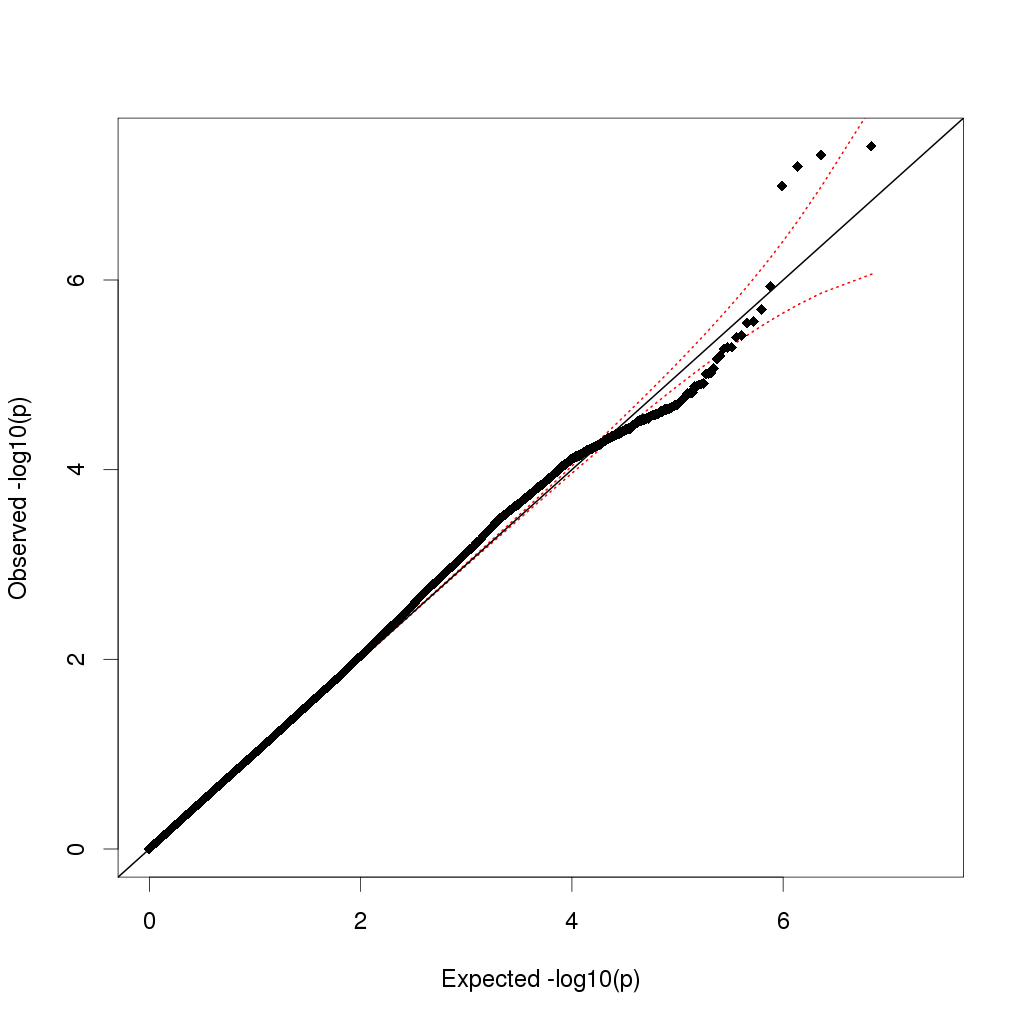


QQ-plots of expected p-values (x-axis) against observed p-values (y-axis) for analyses of (clockwise from top left) large artery stroke (age-at-onset informed), large artery stroke (uninformed), small vessel stroke (uninformed), small vessel stroke (age-at-onset informed). Lambda values for each plot are given in Table S4.
