# Supplementary material for: A Novel MMP12 Locus Is Associated with Large Artery Atherosclerotic Stroke Using a Genome-Wide Age-at-Onset Informed Approach
Source: PLoS Genet. 2014 Jul 31;10(7):e1004469. doi: 10.1371/journal.pgen.1004469 (PMC4117446; doi:10.1371/journal.pgen.1004469)

**Figure S3 – Forest plot of SNP effects for rs660599 in the large artery atherosclerotic stroke replication populations**

ASGC, the Australian Stroke Genetics collaboration; deCODE, deCODE genetics; GEOS, the Genetics of early onset stroke study; HVH, the heart and vascular health study; ISGS/SWISS, the Ischaemic stroke genetics study / Siblings with Ischaemic stroke study; MGH-GASROS, Massachusetts General Hospital – Genetics affecting stroke risk and outcome. PROMISe, Prognostic modeling in ischaemic stroke study; RACE, Risk Assessment of Cerebrovascular Events study
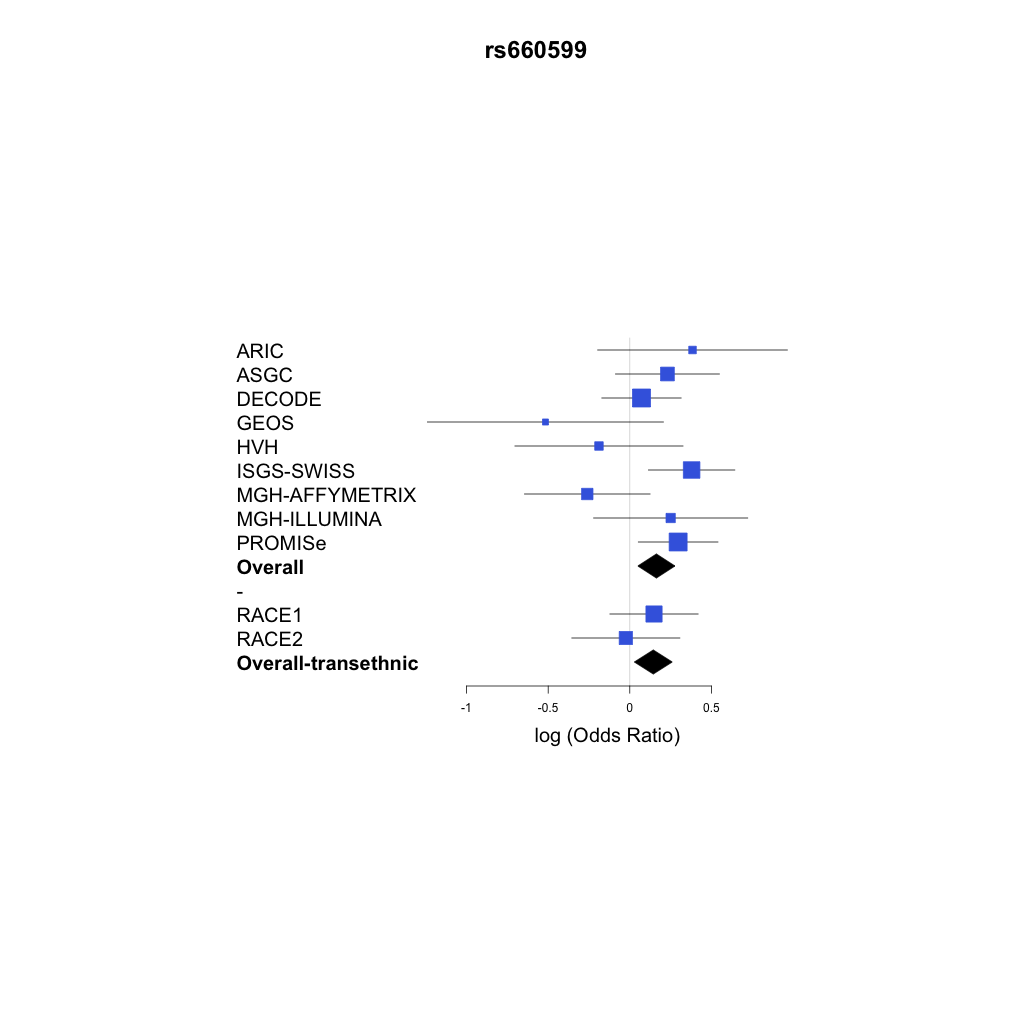

Supplement: Figure S3 — Forest plot of SNP effects for rs660599 in the large artery atherosclerotic stroke replication populations. ASGC, the Australian Stroke Genetics collaboration; deCODE, deCODE genetics; GEOS, the Genetics of early onset stroke study; HVH, the heart and vascular health study; ISGS/SWISS, the Ischaemic stroke genetics study/Siblings with Ischaemic stroke study; MGH-GASROS, Massachusetts General Hospital – Genetics affecting stroke risk and outcome. PROMISe, Prognostic modeling in ischaemic stroke study; RACE, Risk Assessment of Cerebrovascular Events study. (DOCX) [file pgen.1004469.s003.docx]
