# Supplementary material for: A Novel MMP12 Locus Is Associated with Large Artery Atherosclerotic Stroke Using a Genome-Wide Age-at-Onset Informed Approach
Source: PLoS Genet. 2014 Jul 31;10(7):e1004469. doi: 10.1371/journal.pgen.1004469 (PMC4117446; doi:10.1371/journal.pgen.1004469)
Supplement: Table S1 — Results from RegulomeDB, showing the evidence that SNPs in the associated MMP12 region have a regulatory function. Scores indicate the following degrees of evidence: Score 2b, TF binding + any motif + DNase Footprint + DNase peak; Score 4, TF binding + DNase peak; Score 5, TF binding or DNase peak; Score 6, other; “No data” indicates that RegulomeDB holds no information about the given SNP, meaning there currently exists no evidence to suggest that the SNP has a regulatory function. In some cases this may indicate that the SNP falls within a protein-coding region. SNP, single nucleotide polymorphism. (DOCX) [file pgen.1004469.s005.docx]

Table S1 - Results from RegulomeDB, showing the evidence that SNPs in the associated *MMP12* region have a regulatory function

| Linkage Disequilibrium with rs660599 | SNP | p-value | Evidence from RegulomeDB |
| --- | --- | --- | --- |
| **r^2^=1** | rs615098 | 2.9x10^-7^ | No data |
|  | rs626750 | 1.9x10^-7^ | No data |
|  | **rs586701** | **1.8x10^-7^** | **Score 2b: likely to affect binding** |
|  | rs644885 | 2.8x10^-7^ | Score 6: minimal binding evidence |
|  | rs654600 | 2.3x10^-7^ | No data |
|  | rs660599 | 2.5x10^-7^ | No data |
|  | rs674546 | 2.7x10^-7^ | No data |
| **r^2^=0.99** | rs608194 | 2.2x10^-7^ | No data |
|  | rs662558 | 1.4x10^-7^ | Score 6: minimal binding evidence |
| **0.60 < r^2^ < 0.64** | rs116187470 | 2.2x10^-6^ | Score 6: minimal binding evidence |
|  | rs114176245 | 5.5x10^-6^ | No data |
|  | rs17361668 | 7.0x10^-6^ | Score 4: minimal binding evidence |
|  | rs72981675 | 7.5x10^-6^ | No data |
|  | rs72981680 | 7.2x10^-6^ | Score 5: minimal binding evidence |
|  | rs72981683 | 4.7x10^-6^ | Score 5: minimal binding evidence |
|  | rs72981684 | 6.6x10^-6^ | No data |
|  | rs737693 | 6.6x10^-6^ | No data |
|  | rs72981698 | 6.6x10^-6^ | Score 6: minimal binding evidence |
|  | rs72983508 | 6.6x10^-6^ | Score 5: minimal binding evidence |
|  | rs72983513 | 5.7x10^-6^ | Score 6: minimal binding evidence |
|  | rs28381684 | 6.3x10^-6^ | Score 6: minimal binding evidence |
|  | rs17368582 | 4.6x10^-6^ | Score 6: minimal binding evidence |
|  | rs17368659 | 7.3x10^-6^ | No data |
|  | **rs2276109** | 6.6x10^-6^ | **Score 2b: likely to affect binding** |
|  | rs17368814 | 7.5x10^-6^ | Score 4: minimal binding evidence |

Scores indicate the following degrees of evidence: Score 2b, TF binding + any motif + DNase Footprint + DNase peak; Score 4, TF binding + DNase peak; Score 5, TF binding or DNase peak; Score 6, other; “No data” indicates that RegulomeDB holds no information about the given SNP, meaning there currently exists no evidence to suggest that the SNP has a regulatory function. In some cases this may indicate that the SNP falls within a protein-coding region. SNP, single nucleotide polymorphism.
