# Supplementary material for: A Novel MMP12 Locus Is Associated with Large Artery Atherosclerotic Stroke Using a Genome-Wide Age-at-Onset Informed Approach
Source: PLoS Genet. 2014 Jul 31;10(7):e1004469. doi: 10.1371/journal.pgen.1004469 (PMC4117446; doi:10.1371/journal.pgen.1004469)
Supplement: Table S2 — Expanded set of populations used to generate SNPs with p<0.05 to evaluate the age-at-onset informed approach. ARIC, The Atherosclerosis Risk in Communities study; ASGC, Australian Stroke Genetics Collaborative; CHS, Cardiovascular Health Study; FHS, Framingham Heart Study; HPS, Heart Protection Study; HVH, The Heart and Vascular Health Study; ISGS/SWISS, The Ischemic Stroke Genetics Study/Sibling with Ischaemic Stroke Study; MGH-GASROS, The MGH Genes Affecting Stroke Risk and Outcome Study; WTCCC2-Germany, The Wellcome Trust Case-Consortium II Munich; WTCCC2-UK, The Wellcome Trust Case-Consortium II UK; RACE, Risk Assessment of Cerebrovascular Events Study, Pakistan. (DOCX) [file pgen.1004469.s006.docx]

Table S2 – Expanded set of populations used to generate SNPs with p<0.05 to evaluate the age-at-onset informed approach

| Study Population | Number of cases | Number of CE cases | Number of LAA cases | Number of SVD cases | Number of controls |
| --- | --- | --- | --- | --- | --- |
| WTCCC2-Germany | 1,174 | 330 | 346 | 106 | 797 |
| WTCCC2-UK | 2,374 | 460 | 498 | 474 | 5,175 |
| Belgium-immunochip | 396 | 147 | 57 | 49 | 319 |
| Germany-immunochip | 421 | 127 | 101 | 8 | 2,355 |
| Krakow-immunochip | 384 | 119 | 33 | 28 | 255 |
| Sweden-immunochip | 796 | 246 | 56 | 183 | 997 |
| UK-immunochip | 867 | 130 | 152 | 257 | 1,790 |
| PROMISe-immunochip | 556 | - | 324 | 232 | 1,145 |
| ARIC | 385 | 93 | 31 | 63 | 8,803 |
| ASGC | 1,162 | 240 | 421 | 310 | 1,244 |
| CHS | 454 | 147 | - | 73 | 2,817 |
| deCODE | 2,391 | 399 | 255 | 240 | 26,970 |
| FHS | 171 | 48 | - | - | 4,164 |
| GEOS | 448 | 90 | 37 | 54 | 498 |
| HPS | 578 | - | - | - | 468 |
| HVH | 566 | 88 | 61 | 173 | 1,290 |
| ISGS/SWISS | 1,070 | 247 | 229 | 201 | 2,329 |
| MGH-GASROS | 516 | 169 | 95 | 38 | 1,202 |
| Milano | 366 | 64 | 73 | 25 | 407 |
| Rotterdam | 367 | - | - | - | 5,396 |
| Total | 15,442 | 3,144 | 2,769 | 2,514 | 68,421 |

ARIC, The Atherosclerosis Risk in Communities study; ASGC, Australian Stroke Genetics Collabarative; CHS, Cardiovascular Health Study; FHS, Framingham Heart Study; HPS, Heart Protection Study; HVH, The Heart and Vascular Health Study; ISGS/SWISS, The Ischemic Stroke Genetics Study/Sibling with Ischaemic Stroke Study; MGH-GASROS, The MGH Genes Affecting Stroke Risk and Outcome Study; WTCCC2-Germany, The Wellcome Trust Case-Consortium II Munich; WTCCC2-UK, The Wellcome Trust Case-Consortium II UK; RACE, Risk Assessment of Cerebrovascular Events Study, Pakistan.
