# Supplementary material for: A Novel MMP12 Locus Is Associated with Large Artery Atherosclerotic Stroke Using a Genome-Wide Age-at-Onset Informed Approach
Source: PLoS Genet. 2014 Jul 31;10(7):e1004469. doi: 10.1371/journal.pgen.1004469 (PMC4117446; doi:10.1371/journal.pgen.1004469)
Supplement: Table S3 — Age and gender distributions of populations. ARIC, The Atherosclerosis Risk in Communities study; ASGC, Australian Stroke Genetics Collaborative; CHS, Cardiovascular Health Study; FHS, Framingham Heart Study; HPS, Heart Protection Study; HVH, The Heart and Vascular Health Study; ISGS/SWISS, The Ischemic Stroke Genetics Study/Sibling with Ischaemic Stroke Study; MGH-GASROS, The MGH Genes Affecting Stroke Risk and Outcome Study; WTCCC2-Germany, The Wellcome Trust Case-Consortium II Munich; WTCCC2-UK, The Wellcome Trust Case-Consortium II UK; RACE, Risk Assessment of Cerebrovascular Events Study, Pakistan. IS, all ischaemic stroke; CE, cardioembolic stroke; LAA, large artery stroke; SVD, small vessel disease. (DOCX) [file pgen.1004469.s007.docx]

**Table S3 – Age and Gender distributions of populations**

|  | Age (mean ± SD) | | | | | Male gender (%) | | | | |
| --- | --- | --- | --- | --- | --- | --- | --- | --- | --- | --- |
|  | IS | SVD | CE | LAA | controls | IS | SVD | CE | LAA | controls |
| WTCCC2-Germany | 66.9 ± 12.9 | 65.9 ± 11.4 | 71.7 ± 12.1 | 65.8 ± 10.8 | 62.7 ± 10.9 | 61.9 | 72.6 | 52.1 | 70.7 | 51.4 |
| WTCCC2-UK | 72.2 ± 12.3 | 70.4 ± 11.7 | 77.0 ± 12.9 | 70.0 ± 10.2 | 52* | 53.7 | 49.8 | 59.4 | 63.3 | 50.5 |
| Milano | 57.4 ± 15.6 | 59.9 ± 15.0 | 56.2 ± 17.3 | 63.5 ± 10.9 | 50.8 ± 8.1 | 62.8 | 68.0 | 56.7 | 76.0 | 87.7 |
| Belgium-iChip | 69.5 ± 13.3 | 67.2 ± 14.9 | 70.7 ± 13.0 | 68.5 ± 10.8 | - | 63.1 | 67.3 | 63.9 | 73.7 | - |
| Germany-iChip | 69.2 ± 13.9 | - | 71.6 ± 14.0 | 69.3 ± 11.3 | - | 57.6 | - | 52.7 | 69.7 | - |
| Krakow-iChip | 69.7 ± 13.5 | 70.7 ± 11.1 | 76.5 ± 10.8 | 69.1 ± 11.0 | - | 55.2 | 42.9 | 54.8 | 66.7 | - |
| Sweden-iChip | 74.5 ± 11.6 | 70.2 ± 12.2 | 78.8 ± 10.0 | 70.6 ± 10.8 | - | 53.6 | 57.4 | 48.8 | 67.9 | - |
| UK-iChip | 70.1 ± 13.2 | 69.7 ± 13.4 | 71.5 ± 10.9 | 69.0 ± 13.3 | - | 51.1 | 50.1 | 60.8 | 52.0 | - |
| ARIC | 57.3 ± 5.3 | - | - | 57.8 ± 4.3 | 54.1 ± 5.7 | 60.3 | - | - | 58.1 | 46.5 |
| ASGC | 72.9 ±  13.2 | - | - | 73.2 ± 12.3 | 66.3 ± 7.5 | 59.2 | - | - | 68.7 | 50.2 |
| deCODE | 73.3 ±  12.1 | - | - | 70.5 ± 9.3 | 57.3 ±  21.4 | 55.0 | - | - | 61.2 | 38.0 |
| GEOS | 41.0 ± 7.0 | - | - | 45.2 ± 2.7 | 39.5 ± 6.7 | 61.4 | - | - | 67.6 | 56.5 |
| HVH | 69.2 ± 8.6 | - | - | 67.9 ± 9.4 | 66.6 ± 9.1 | 33.7 | - | - | 44.3 | 47.7 |
| ISGS/SWISS | 66.5 ± 13.7 | - | - | 64.5 ± 13.6 | 64.7 ± 12.7 | 65.9 | - | - | 62.2 | 46.6 |
| MGH-GASROS (Affymetrix) | 66.5 ± 14.6 | - | - | 65.6 ± 11.6 | 72.2 ± 7.1 | 64.2 | - | - | 67.7 | 49.3 |
| MGH-GASROS (Illumina) | 67.1 ± 14.5 | - | - | 57.9 ± 11.3 | 43.0 ± 7.8 | 60.0 | - | - | 68.6 | 75.8 |
| PROMISe | 62.7 ± 12.2 | - | - | 63.5 ± 10.7 | - | 65.6 | - | - | 68.3 | - |
| RACE | 59.3 ± 11.2 | - | - | 60.4 ± 9.3 | 53.9 ± 9.3 | 53.6 | - | - | 56.1 | 74.1 |

ARIC, The Atherosclerosis Risk in Communities study; ASGC, Australian Stroke Genetics Collabarative; CHS, Cardiovascular Health Study; FHS, Framingham Heart Study; HPS, Heart Protection Study; HVH, The Heart and Vascular Health Study; ISGS/SWISS, The Ischemic Stroke Genetics Study/Sibling with Ischaemic Stroke Study; MGH-GASROS, The MGH Genes Affecting Stroke Risk and Outcome Study; WTCCC2-Germany, The Wellcome Trust Case-Consortium II Munich; WTCCC2-UK, The Wellcome Trust Case-Consortium II UK; RACE, Risk Assessment of Cerebrovascular Events Study, Pakistan. IS, all ischaemic stroke; CE, cardioembolic stroke; LAA, large artery stroke; SVD, small vessel disease.
