# Supplementary material for: A Novel MMP12 Locus Is Associated with Large Artery Atherosclerotic Stroke Using a Genome-Wide Age-at-Onset Informed Approach
Source: PLoS Genet. 2014 Jul 31;10(7):e1004469. doi: 10.1371/journal.pgen.1004469 (PMC4117446; doi:10.1371/journal.pgen.1004469)
Supplement: Table S4 — Genomic inflation (λ) rates for discovery populations for age-at-onset informed and uninformed approaches. IS, all ischaemic stroke; CE, cardioembolic stroke; LAA, large artery stroke; SVD, small vessel disease. (DOCX) [file pgen.1004469.s008.docx]

**Table S4 – Genomic inflation (**$\boldsymbol{\lambda}$**) rates for discovery populations for age-at-onset informed and uninformed approaches**

|  | Age-at-onset informed analysis | | | | Uninformed analysis | | | |
| --- | --- | --- | --- | --- | --- | --- | --- | --- |
|  | IS | SVD | CE | LAA | IS | SVD | CE | LAA |
| WTCCC2-Germany | 1.027 | 1.006 | 1.003 | 1.012 | 1.025 | 1.005 | 1.003 | 1.013 |
| WTCCC2-UK | 1.036 | 1.015 | 1.018 | 1.018 | 1.032 | 1.014 | 1.015 | 1.016 |
| Milano | 0.971 | 1.045 | 1.007 | 1.020 | 0.969 | 1.044 | 1.004 | 1.017 |
| Belgium-iChip | 1.009 | 0.999 | 1.028 | 1.000 | 1.022 | 0.997 | 1.026 | 1.010 |
| Germany-iChip | 1.150 | - | 1.184 | 1.145 | 1.152 | - | 1.183 | 1.147 |
| Krakow-iChip | 0.997 | 1.053 | 1.013 | 1.043 | 0.998 | 1.051 | 1.010 | 1.045 |
| Sweden-iChip | 1.048 | 1.035 | 1.033 | 1.014 | 1.072 | 1.029 | 1.033 | 1.017 |
| UK-iChip | 1.193 | 1.072 | 1.075 | 1.034 | 1.198 | 1.072 | 1.073 | 1.037 |
| **Overall (lambda)** | 1.086 | 1.016 | 1.040 | 1.054 | 1.080 | 1.014 | 1.043 | 1.051 |
| **Overall (lambda_1000_)** | 1.010 | 1.008 | 1.014 | 1.022 | 1.009 | 1.002 | 1.013 | 1.021 |

IS, all ischaemic stroke; CE, cardioembolic stroke; LAA, large artery stroke; SVD, small vessel disease.
