# Supplementary material for: A Novel MMP12 Locus Is Associated with Large Artery Atherosclerotic Stroke Using a Genome-Wide Age-at-Onset Informed Approach
Source: PLoS Genet. 2014 Jul 31;10(7):e1004469. doi: 10.1371/journal.pgen.1004469 (PMC4117446; doi:10.1371/journal.pgen.1004469)
Supplement: Table S5 — Number of SNPs used in evaluation of age-at-onset informed approach. IS, all ischaemic stroke; CE, cardioembolic stroke; LAA, large artery stroke; SVD, small vessel disease. (DOCX) [file pgen.1004469.s009.docx]

**Table S5 – number of SNPs used in evaluation of age-at-onset informed approach**

| **SNP selection criteria** | **CE** | **LAA** | **SVD** | **IS** |
| --- | --- | --- | --- | --- |
| p<0.05 | 8,527 | 9,290 | 7,639 | 8,379 |
| p<0.01 | 2,563 | 2,843 | 2,176 | 2,749 |
| p<0.005 | 1,501 | 1,607 | 1,253 | 1,654 |
| p<0.001 | 424 | 447 | 307 | 508 |
| p<0.0005 | 244 | 265 | 178 | 308 |
| p<0.0001 | 61 | 85 | 41 | 95 |
| p<0.00005 | 32 | 50 | 26 | 59 |

IS, all ischaemic stroke; CE, cardioembolic stroke; LAA, large artery stroke; SVD, small vessel disease.
