# Supplementary material for: A Novel MMP12 Locus Is Associated with Large Artery Atherosclerotic Stroke Using a Genome-Wide Age-at-Onset Informed Approach
Source: PLoS Genet. 2014 Jul 31;10(7):e1004469. doi: 10.1371/journal.pgen.1004469 (PMC4117446; doi:10.1371/journal.pgen.1004469)
Supplement: Text S2 — Liability threshold models. (DOCX) [file pgen.1004469.s011.docx]

**Text S2 - Liability Threshold Models**

Using the notation from Zaitlen et al, [[1](#_ENREF_1)] if we denote a continuous unobserved quantitative trait called the disease liability as:

$$\varphi=\sum_{j=1}^{J} c_{j}\left( t_{j}-\bar{t_{j}} \right)+m+\varepsilon(1)$$

where $\varepsilon=\gamma g+N(0,1)$ and *g* is the genetic component, then an individual is a case (*z=1*) if and only if $\varphi\geq0$ and is a control (*z=0*) otherwise. Here, $c_{j}$ is a parameter estimating the effect of a given covariate *j* on the liability scale. Covariates with strong effects, such as body-mass-index in type 2 diabetes or smoking status in lung cancer would be larger than weaker covariates. $m$ denotes the disease prevalence *p* at the covariate mean $\bar{t_{j}}$ under a normal cumulative distribution function $( \Phi\left( -m \right)=p)$.

The lifetime risk of ischaemic stroke at different ages has been estimated using individuals from the Framingham Heart Study. [[2](#_ENREF_2),[3](#_ENREF_3)] The results showed that ischaemic stroke is considerably more prevalent at older age ranges. From an index of 55 years of age, risk of ischaemic stroke is estimated to be 2.1%, 6.4% and 12.4% before age 65, 75 and 85 years respectively.

Using the above estimates of prevalence at stroke at given age-at-onset thresholds, we can estimate the liability threshold parameters in (1). We denote the following as the prevalence at covariate value $t_{j}$, given liability parameters $c_{j}$ and *m:*

$$f\left( t_{j} \right)= \Phi{(-c}_{j}\left( t_{j}-\bar{t_{j}} \right)-m)$$

Entering age-at-onset prevalence values into the equation gives the following:

$$f\left( 65 \right)=0.021 \Rightarrow\Phi{(-c}_{j}\left( 10 \right)-m)=0.021$$

$$f\left( 75 \right)=0.064\Rightarrow\Phi{(-c}_{j}\left( 20 \right)-m)=0.064$$

$$f\left( 85 \right)=0.124\Rightarrow\Phi{(-c}_{j}\left( 30 \right)-m)=0.124$$

This can then be solved analytically, giving $m=-2.45$*,* $c_{j}=0.044$*,* meaning the liability parameters for all ischaemic stroke cases can be calculated using:

$$\varepsilon_{j}=-0.044\left( t_{j}-55 \right)+2.45$$

Similarly for the ischaemic stroke subtypes, we assume that 20% of ischaemic stroke cases are cardioembolic, large artery or small vessel stroke. Following the same procedure, we obtain the following liability model:

$$\varepsilon_{j}=-0.035\left( t_{j}-55 \right)+2.97$$

Posterior liabilities for each individual can then be calculated as follows, conditioned on the point on the standard normal distribution (considering the covariate) at which they had a stroke event:

$$E\left( \varepsilon|z,t \right)=\frac{\int_{-c\left( t-\bar{t} \right)-m}^{\infty} \varepsilon\frac{1}{\sqrt{2\pi}}e^{\left( \frac{-\varepsilon^{2}}{2} \right)}d\varepsilon}{\int_{-c\left( t-\bar{t} \right)-m}^{\infty} \frac{1}{\sqrt{2\pi}}e^{\left( \frac{-\varepsilon^{2}}{2} \right)}d\varepsilon}, if z=1$$

$$E\left( \varepsilon|z,t \right)=\frac{\int_{\infty}^{-c\left( t-\bar{t} \right)-m} \varepsilon\frac{1}{\sqrt{2\pi}}e^{\left( \frac{-\varepsilon^{2}}{2} \right)}d\varepsilon}{\int_{\infty}^{-c\left( t-\bar{t} \right)-m} \frac{1}{\sqrt{2\pi}}e^{\left( \frac{-\varepsilon^{2}}{2} \right)}d\varepsilon}, if z=0$$

Regression is then performed on $E\left( \varepsilon|z,t \right)$, calculating the number of samples multiplied by the squared correlation between the expected genotype dosage *g* (from imputation) and $E\left( \varepsilon|z,t \right).$

*Post-hoc association analysis*

In order to determine the association of the associated region across different age-at-onset thresholds, we performed logistic regression per centre, including ancestry-informative principal components where relevant. Overall effects across all centres were determined after meta-analysis using a fixed effects inverse-variance weighted approach, as implemented in METAL. [[4](#_ENREF_4)]

*References*

1. Zaitlen N, Lindstrom S, Pasaniuc B, Cornelis M, Genovese G, et al. (2012) Informed conditioning on clinical covariates increases power in case-control association studies. PLoS Genet 8: e1003032.

2. Seshadri S, Wolf PA (2007) Lifetime risk of stroke and dementia: current concepts, and estimates from the Framingham Study. Lancet Neurol 6: 1106-1114.

3. Seshadri S, Beiser A, Kelly-Hayes M, Kase CS, Au R, et al. (2006) The lifetime risk of stroke: estimates from the Framingham Study. Stroke 37: 345-350.

4. Willer CJ, Li Y, Abecasis GR (2010) METAL: fast and efficient meta-analysis of genomewide association scans. Bioinformatics 26: 2190-2191.
